# Vitamin D Supplementation and Its Interaction with Common Medications: Impact on Serum Levels and Quality of Life in Adults with Comorbidities

**DOI:** 10.3390/ph18111727

**Published:** 2025-11-13

**Authors:** Fernando Lopez-Carmona, Antonio Toro-Ruiz, Celia Piquer-Martinez, Manuel Gomez-Guzman, Francisco Javier Ferreira-Alfaya, Maria Isabel Valverde-Merino, Francisco Rivas-Garcia, Maria Jose Zarzuelo

**Affiliations:** 1 Department of Pharmacy and Pharmaceutical Technology, School of Pharmacy, University of Granada, 18071 Granada, Spain; galaxdar@gmail.com (F.L.-C.); atoru.6@gmail.com (A.T.-R.); celiapiquermartinez@gmail.com (C.P.-M.); misabelvalverdemerino@gmail.com (M.I.V.-M.); mjzarzuelo@ugr.es (M.J.Z.); 2 Department of Pharmacology, School of Pharmacy, University of Granada, 18071 Granada, Spain; mgguzman@ugr.es; 3 Center for Biomedical Research (CIBM), 18012 Granada, Spain; 4 Instituto de Investigación Biosanitaria de Granada, ibs.GRANADA, 18012 Granada, Spain; 5 Municipal Health and Consumer Unit, Guadix City Council, 18500 Guadix, Spain

**Keywords:** vitamin D, drug interaction, supplement, quality of life, medication

## Abstract

**Background/Objectives**: Vitamin D deficiency is highly prevalent worldwide and is associated with multiple comorbidities and pharmacological treatments that may interfere with its metabolism. Evidence on the effect of supplementation across different drug user groups remains limited. **Methods**: A prospective study was conducted across community pharmacies over twelve months. Baseline socio-demographic, serum 25(OH)D concentration, quality of life (QoL), lifestyle habits, and medication use were collected. Participants received vitamin D supplementation for 12 months. Changes in vitamin D status and QoL were analyzed according to medication use. Logistic regression identified predictors of achieving adequate serum vitamin D levels (>30 ng/mL). Statistical significance was set at *p* < 0.05. **Results**: At baseline, 87.2% of 210 participants had insufficient or deficient vitamin D levels. After supplementation, mean serum vitamin D increased significantly from 21.3 ± 8.2 to 32.1 ± 12.6 ng/mL (*p* < 0.001), and QoL scores improved from 68.6 ± 18.7 to 77.8 ± 18.5 (*p* < 0.001). Dietary intake of vitamin D–rich foods and outdoor activity also increased. Supplementation improved vitamin D status among users of benzodiazepines, proton pump inhibitors, beta-blockers, statins, levothyroxine, metformin, and angiotensin-converting enzyme inhibitors, but not among corticosteroid, nonsteroidal anti-inflammatory drugs, or vitamin K antagonist. Multivariate analysis confirmed adherence as a strongest predictor of vitamin D adequacy (OR = 15.31, 95% CI = 2.90–80.75), while corticosteroid therapy, diabetes, and hypercholesterolemia were negatively associated. **Conclusions**: Vitamin D supplementation effectively corrected deficiency and improved QoL, but its efficacy varied according to comorbidities and medication use. Personalized supplementation strategies, emphasizing adherence and considering pharmacological profiles, may optimize outcomes. Further studies should explore mechanistic drug–nutrient interactions and long-term clinical implications.

## 1. Introduction

Vitamin D is a fat-soluble prohormone known primarily for its fundamental role in calcium and phosphate homeostasis, essential for proper bone metabolism and musculoskeletal health [1]. Beyond this classical function, its active metabolite (calcitriol) acts as a powerful pleiotropic regulator, implicated in immune modulation, cardiovascular function, and cellular differentiation [2,3,4]. Deficiency in vitamin D has been associated with an increased risk of chronic diseases, including cardiovascular disease, type 2 diabetes, certain cancers, and autoimmune disorders [5,6,7,8,9,10,11,12,13,14].

Despite this broad clinical relevance, vitamin D deficiency (VDD) remains highly prevalent worldwide, generally defined by serum 25(OH)D level below 20 ng/mL. In Spain, recent reports suggest that up to 80% of individuals over the age of 65 have suboptimal levels, highlighting a major public health gap [15,16,17].

From an integrative health perspective, it is vital to recognize that Vitamin D transcends mineral homeostasis; its role as an immune modulator is crucial [18], and its involvement has been suggested in mood regulation and the perception of chronic pain [19,20], all of which directly impact Quality of Life (QoL).

Pharmacological supplementation is widely recommended to correct VDD. However, therapeutic adherence remains suboptimal (estimated at approximately 20%) due to complex dosing and poor patient education regarding sun exposure and dietary sources [21,22]. Community pharmacists are uniquely positioned to address these barriers. As accessible healthcare professionals, they can provide patient-centered education, monitor adherence, and collaborate with primary care providers to optimize supplementation strategies in chronic disease management [23,24,25,26,27,28,29].

Given the high prevalence of VDD and the documented challenges in adherence and patient awareness [30,31,32], this study aims to evaluate whether a structured pharmacist-led intervention—focused on education, supplementation guidance, and regular follow-up—can improve serum 25(OH)D levels and enhance self-perceived QoL in a southern Spanish population.

Scientific literature has shown that there are a number of interactions between vitamin D supplements and drugs which have shown variations in plasma vitamin D (VD) concentration (Table 1) [33,34]:

Despite the known prevalence of VDD among multimorbid patients and the reported in vitro and in vivo interactions with common prescription drugs, few clinical studies have simultaneously assessed these variables within a primary care setting, where patient follow-up and adherence are key. Therefore, the primary objective of this study was to evaluate the impact of vitamin D supplementation on serum 25(OH)D levels and QoL in adults with comorbidities, with a specific focus on characterizing the differential effect of concomitant chronic pharmacological treatments and assessing adherence through supervised pharmaceutical care.

## 2. Results

A total of 210 participants completed the study, of whom 77.8% were women and 22.2% men, with a mean age of 58.52 ± 14.15 years. Regarding socioeconomic status, 39.2% were retired, and 49.5% had attained a university-level education. The most prevalent comorbidities were hypertension (46.6%) and hypercholesterolemia (29.2%). Fifteen point two percent (15.2%) of the participants presented no underlying pathology. At baseline, VDD was highly prevalent: 50.6% of participants were classified as deficient (<20 ng/mL), 36.6% as insufficient (20–29.99 ng/mL), and only 12.8% had adequate levels (≥30 ng/mL) (Table 2).

Monthly VD supplementation was the most common regimen (71.9% of cases), and overall adherence was high, with 78.2% of the participants adhering to correct protocol at the 12-month follow-up. After 12 months of supplementation, serum 25(OH)D concentrations significantly increased from 21.3 ± 8.2 ng/mL to 32.1 ± 12.6 ng/mL (*p* < 0.001). This biochemical improvement was paralleled by a significant increase in QoL scores (from 68.6 ± 18.7 to 77.8 ± 18.5 points out of 100, *p* < 0.001). Body mass index (BMI) remained stable throughout the study period (25.3 ± 4.2 vs. 25.2 ± 4.2 kg/m^2^, *p* = 0.848).

Changes in lifestyle habits were also noted. Participants reported an increased frequency of outdoor sports activity (from 2.3 ± 2.9 to 3.3 ± 3.9 h per week, *p* = 0.003), while daily walking time showed a non-significant increasing trend (*p* = 0.093). Dietary analysis revealed increased consumption of several vitamin D–rich foods, including avocado (*p* < 0.001), tuna (*p* = 0.003), salmon (*p* = 0.010), eggs (*p* < 0.001), and mushrooms (*p* = 0.003). Sardine/anchovy intake did not change significantly (*p* = 0.308). Paradoxically, the consumption of vitamin D–fortified dairy products decreased significantly (*p* = 0.001) (Table 3).

The response to supplementation varied significantly based on concomitant pharmacological treatment. Specifically, participants who were not taking any medication reduced their deficiency from 40.6% to 21.8% and increased their adequate level category from only 6.3% to 46.9% after 12 months of VD supplementation (*p* < 0.001). Significant improvements in VDD categories were observed among users of benzodiazepines (*p* = 0.006), Proton Pump Inhibitors (PPIs) (*p* = 0.042), beta-blockers (*p* = 0.009), levothyroxine (*p* = 0.003), biguanides (*p* = 0.039), xanthine oxidase inhibitors (*p* = 0.020), statins (*p* = 0.003), and angiotensin-converting enzyme (ACE) inhibitors (*p* = 0.032). For these groups, supplementation successfully shifted patients from deficient or insufficient status towards adequacy. In contrast, patients treated with corticosteroids, nonsteroidal anti-inflammatory drugs (NSAIDs), vitamin K antagonists, or inhaled therapies for asthma/COPD showed no significant changes in VDD categories after supplementation. For instance, corticosteroid users remained predominantly in the deficient range, despite supplementation (*p* = 0.661) (Table 4).

Analysis of mean serum 25(OH)D levels confirmed the previous finding. The non-medicated group showed a significant increase mean VD levels (baseline: 21.35 ± 7.17 ng/mL: 12 months: 30.11 ± 11.97 ng/mL (*p* < 0.050). Significant increases in mean serum 25(OH)D levels were also documented in patients using: benzodiazepines (*p* < 0.001), PPIs (*p* < 0.050), beta-blockers (*p* < 0.001), levothyroxine (*p* < 0.050), biguanides (*p* < 0.050), xanthine oxidase inhibitors (*p* < 0.050), statins (*p* < 0.001), ACE Inhibitors (*p* < 0.050), acetaminophen (*p* < 0.050), calcium channel blockers (*p* < 0.050), and antihistamines (*p* < 0.050). No significant changes in mean 25(OH)D levels were observed in patients taking NSAIDs, vitamin K antagonists, inhalers for asthma/COPD, ezetimibe, thiazide and loop diuretics, corticosteroids, serotonin reuptake inhibitors, angiotensin II antagonists, or insulin. QoL scores significantly improved after supplementation only in the non-medicated group and those receiving benzodiazepines, beta-blockers, and statins (all *p* < 0.050) (Table 5).

Baseline Comparisons (Between-Group): At baseline, significant differences in serum 25(OH)D levels were observed only when comparing patients taking diuretics (26.03 ± 11.34 ng/mL) and angiotensin II antagonists (25.73 ± 13.21 ng/mL) against the ‘Other drugs’ group (*p* = 0.004 and *p* = 0.027, respectively). However, significant differences in QoL scores were observed between almost all drug categories and the non-medicated group at both baseline and 12 months (Table 4), indicating a strong initial impact of chronic medication status on QoL.

Logistic regression analysis identified significant predictors of adequate VD status. Post-supplementation, adherence emerged as a strong positive predictor of adequate 25(OH)D levels (OR = 15.31; 95% CI: 2.90–80.75). At 12 months, the consumption of NSAIDs (OR = 0.47; 95% CI: 0.40–0.56), inhalers for asthma/COPD (OR = 0.47; 95% CI: 0.40–0.56), and corticosteroids (OR = 0.62; 95% CI: 0.40–0.97) were significantly associated with a reduced likelihood of achieving adequate VD status. Conversely, PPI consumption (OR = 1.83; 95% CI: 1.04–3.23) and no pathologies (OR = 4.00; 95% CI: 1.29–11.51) were positively associated with adequate status. Furthermore, diabetes (OR = 0.20; 95% CI: 0.06–0.67) and hypercholesterolemia (OR = 0.25; 95% CI: 0.09–0.69) were strongly and negatively associated with adequate 25(OH)D levels after the intervention. Baseline predictors of adequate 25(OH)D levels were more complex: Diuretics (OR = 4.12; 95% CI: 1.26–13.47), Angiotensin II Antagonists (OR = 4.70; 95% CI: 1.28–17.26), and Insulin (OR = 7.05; 95% CI: 1.09–45-78) were positively associated, while Beta-blockers, Xanthine Oxidase Inhibitors, Calcium Channel Blockers, and Antihistamines were negatively associated (OR < 1 for all), indicating that many of these drug-related associations were attenuated or reversed following the 12-month supplementation protocol (Table 6).

## 3. Discussion

The present study demonstrates that VD supplementation significantly improved mean serum 25(OH)D concentration and self-perceived QoL in a cohort with a high prevalence of deficiency and comorbidities. After 12 months of treatment, mean serum concentrations increased from insufficient levels to values within the adequate range, confirming the effectiveness of supplementation protocol in this vulnerable population.

These findings are consistent with prior studies demonstrating the efficacy of supplementation in correcting VDD in adults. VDD remains widespread in Europe, with serum concentrations below 12 ng/mL and 20 ng/mL observed in approximately 13% and 40% of the population, respectively [35]. While promoting lifestyle modifications such as diet and sun exposure is important, these measures are often insufficient to ensure adequacy. Fortification programs in the United States, Canada, Finland, and India have proven effective and may represent a necessary public health strategy in Europe as well [35]. In our cohort, participants increased intake of vitamin D–rich foods (eggs, fatty fish, mushrooms) and outdoor activity, potentially contributing synergistically to improved VD status. The observed decline in fortified dairy intake may reflect dietary preferences or counseling, but overall, lifestyle modifications complemented the effects of supplementation. Similar to previous reports, outdoor activity was relevant, and evidence suggests that combining VD with exercise enhances functional outcomes, including walking speed in older adults with deficiency [36].

We observed a significant 8-point increase in QoL scores after supplementation, consistent with studies linking higher VD levels to improved well-being, particularly in chronic disease populations [37,38,39,40]. For example, in a large trial of individuals with irritable bowel syndrome, VD supplementation was associated with improved QoL and higher serum concentrations [41]. In our cohort, QoL improvements were particularly pronounced among benzodiazepine, beta-blocker, and statin users, corroborating evidence that VD supports musculoskeletal and mental health. Conversely, users of corticosteroids and NSAIDs did not experience significant QoL gains, likely reflecting the chronic burden of underlying disease or reduced biological response to supplementation.

One key novelty of this work was the evaluation of VD response according to concurrent pharmacological treatment. Supplementation successfully improved VD status among users of benzodiazepines, PPIs, beta-blockers, statins, levothyroxine, biguanides, and ACE inhibitors. Biguanide users, in particular, reached among the highest post-supplementation levels, in line with evidence that VD may improve glycemic control and inflammation in diabetes [10,42,43].

In contrast, corticosteroid, NSAID, and vitamin K antagonist users did not improve significantly. The blunted response among corticosteroid users aligns with literature showing that glucocorticoids accelerate VD catabolism via induction of 24-hydroxylase, thereby impairing calcium absorption and reducing circulating 25(OH)D [44]. NSAID users also showed limited benefit, potentially due to chronic inflammation or pharmacological interference with VD pathways.

These findings regarding other drug groups generally support previous experimental and clinical studies. For example, thiazide diuretics have been associated with increased circulating 25(OH)D in Danish trials [45], and consistently, our cohort showed that diuretic users had higher odds of adequacy at baseline. Statin use has been linked to higher 25(OH)D levels in both randomized trials and observational studies [46,47,48], consistent with our finding of significant improvement post-supplementation. Conversely, calcium channel blocker use has been associated with reduced 25(OH)D via CYP3A4 inhibition [49], in our analysis, users showed lower baseline odds of adequacy and only modest improvement with supplementation. Similarly, ACE inhibitors have been linked to reduced 25(OH)D levels in observational studies [50,51]; however, in our population, supplementation shifted many patients from deficient to adequate categories (*p* = 0.032), suggesting the intervention can overcome the pharmacological antagonism.

Regarding specific pharmacological classes, while some cross-sectional studies reported lower 25(OH)D concentrations in vitamin K antagonism users [52,53], we observed no significant effects on VDD status. Likewise, benzodiazepine use has been inconsistently associated with lower VD in cross-sectional analyses [54], yet supplementation proved effective in our cohort. Selective serotonin reuptake inhibitors have also been linked to lower VD status [54,55], though we found no significant changes in the post-supplementation category. Inhaled corticosteroids (ICS) present another complex interaction; some studies suggest negative effects on bone metabolism and modest increases in fracture risk [44,56]. Our logistic regression findings indicated that patients using ICS had significantly lower odds (OR = 0.47) of achieving adequate VDD status post-supplementation, reinforcing concerns of a reduced therapeutic response and accelerated catabolism in this subgroup.

Beyond bone health, VD sufficiency has been linked to cardiovascular and metabolic benefits. Supplementation may reduce cardiac hypertrophy, improve lipid profiles, and lower atherosclerosis risk [42,57,58,59]. In our study, patients who achieved adequate 25(OH)D levels post-supplementation were significantly less likely to present with hypercholesterolemia, consistent with previous reports. Similarly, VD has been shown to improve insulin resistance and inflammatory markers in type 2 diabetes [43]. Our data corroborate these findings, as patients with adequate VD after supplementation were less likely to have diabetes.

Adherence to supplementation emerged as the strongest predictor of achieving adequacy, consistent with prior reports highlighting the pharmacist’s role in supporting compliance [60,61]. Among pharmacological predictors, PPIs were associated with increased odds of adequacy post-intervention, in line with studies reporting no clinically meaningful reduction in VD with chronic PPI use [62]. Conversely, corticosteroid use, diabetes, and hypercholesterolemia were associated with a lower probability of adequacy, emphasizing the need for highly individualized supplementation strategies. In conclusion, our findings confirm the effectiveness of VD supplementation for improving serum levels and QoL, while highlighting variable responses depending on comorbidities and medication use. These results underscore the importance of patient adherence and personalized supplementation strategies in clinical practice.

The strengths of this study include its prospective design, the systematic assessment of both biochemical and lifestyle parameters, and the comprehensive evaluation of medication–vitamin D interactions. Limitations include the observational nature of the analysis, which precludes establishing definitive causal relationships, and the potential influence of unmeasured confounders such as seasonal variation in sun exposure or genetic polymorphisms affecting VD metabolism. Additionally, subgroup analyses for some medications were limited by small sample sizes, thus reducing statistical power.

Our results support the routine evaluation and supplementation of VD in populations at high risk of deficiency, particularly among patients with multiple comorbidities and those receiving medications known to interfere with VD metabolism. Personalized supplementation strategies and adherence reinforcement are essential to optimize outcomes. Future studies should explore the mechanistic basis of drug–vitamin D interactions and assess whether correction of deficiency translates into improved long-term clinical outcomes, such as fracture prevention, metabolic control, and mental health benefits.

## 4. Materials and Methods

### 4.1. Study Design

A multicenter, quasi-experimental, prospective study was conducted using non-probabilistic convenience sampling. The study took place over 12 months (January 2024 to January 2025) in ten community pharmacies located in both urban and rural areas of southern Spain.

Participating pharmacists were previously trained in the detection and management of VDD following the guidelines of the Spanish Society of Endocrinology and Nutrition (SEEN) [17]. The pharmacists administered a previously designed survey instrument.

Participants were provided with scheduled reminders to take VD, along with a monthly video (up to 1.5 min long) containing educational content about VDD, its consequences, and strategies to improve 25(OH)D levels. Additionally, an information sheet was created for primary care physicians (PCPs) requesting their collaboration in providing access to previous lab results and promoting periodic evaluation (every 4–6 months).

### 4.2. Data Collection

The survey instrument used was structured into five main sections: socio-health characteristics, physical activity, diet, medication use, and health status perception. Items were selected and adapted from the European Health Survey in Spain (2020), prioritizing those related to VDD risk factors. Self-perceived QoL was assessed using a Visual Analog Scale (VAS) validated and widely used tools in primary care [63].

A pilot version was tested on 49 patients and reviewed by a multidisciplinary panel of 15 VD experts, who used the Delphi method to reach consensus and refine the questionnaire content [64]. Data collection was conducted at baseline and 12 months. Sociodemographic data were collected only at baseline.

### 4.3. Study Population

Participants were recruited by pharmacists who identified potential symptoms of VDD or noted an active prescription for VD. Potential cases were detected through:

- Review of the electronic health card during medication dispensing.
- Private or mutual fund prescriptions.
- Identification of symptoms or lifestyle habits compatible with VDD during patient interactions.

#### 4.3.1. Inclusion Criteria

- Age > 14 years
- Signed informed consent (parental authorization for minors was required)
- Diagnosis or suspicion of VDD/insufficiency
- Use of medications known to affect VD absorption/metabolism (Table 7)

#### 4.3.2. Exclusion Criteria

- Liver, biliary, renal, or cardiac insufficiency
- Hypoparathyroidism
- History of kidney stones
- Inability to read or speak Spanish
- Lack of a mobile phone or messaging application

**Table 7:** Medications and dosage used.

Group of Drugs	Specific Drug and Dosage
Corticosteroid	Fluticasone 100 mcg, mometasone 50 mcg, budesonide 64 mcg, beclomethasone 50 or 100 mcg
Benzodiazepine	Bromazepam 1.5 or 3 mg, alprazolam 0.5 mg, lorazepam 1 or 2 mg, lormetazepam 1 mg, diazepam 5 mg
Proton pump inhibitor	Pantoprazole 20 mg, esomeprazole 20 or 40 mg, lansoprazole 30 mg, omeprazole 20 or 40 mg
Beta blocker	Bisoprolol 2.5 or 5 mg
NSAID	Ibuprofen 400 or 600 mg, naproxen 550 mg, diclofenac 75 mg, dexketoprofen 25 mg
Levothyroxine	Levothyroxine 25 mg, 50, 88, 100 or 150 mg
Vitamin K antagonists	Acenocumarol 4 mg
Biguanide	Metformin 850 mg
Inhaler asthma/COPD	Formoterol 12 mcg, salbutamol 100 mcg, terbutalin 500 mcg, tiotropium 10 mcg, glycopyrronium 44 mcg
Xanthine oxidase inhibitor	Allopurinol 100 mg
Ezetimibe	Ezetimibe 10 mg
Statin	Rosuvastatin 10 or 20 mg, atorvastatin 10 or 40 or 80 mg, simvastatin 10 or 20 mg, pravastatin 10 mg, pitavastatin 4 mg
Diuretic	Hydrochlorothiazide 12.5 mg or 25 mg, furosemide 40 mg
Serotonin reuptake inhibitor	Sertraline 50 or 100 mg, fluoxetine 20 mg
Antagonists of the angiotensin II	Olmesartan 40 mg, valsartan 80 or 160 mg, losartan 25 mg, telmisartán 20 mg, irbersartan 150 mg
ACE inhibitor	Enalapril 20 mg, ramipril 10 mg, perindopril 4 mg
Acetaminophen	Acetaminophen 500, 650 or 1000 mg
Insulin	Insulin glargine 100 unidades/mL, modern insulin 100 unidades/ml
Calcium channel blocker	Manodipine 10 mg, amlodipine 5 or 10 mg
Antihistamine	Loratadine 10 mg, cetirizine 10 mg, bilastine 20 mg

NSAID: Non-Steroidal Anti-Inflammatory Drugs; ACE: Angiotensin-Converting Enzyme.

### 4.4. Sample Size Calculation

The sample size was calculated using the formula:

n = N × Z2 × p (1 − p)/(N −1) + e2 + Z2 × (1 − p)

where n is the sample size, N is the target population, Z is the confidence level (95%), p is the estimated prevalence of VDD (37%), and e is the margin of error (7%).

Based on a reference population of 919,700 people in southern Spain, an expected variability of 50%, and a dropout rate of 20%, the required sample size was estimated at 197 participants.

### 4.5. Study Outcomes

Main variables included:

- Sociodemographic data
- Dietary intake of VD per week
- Serum 25(OH)D levels
- Medication use and associated conditions
- Self-perceived Quality of life (VAS 0–100)
- Weekly physical activity
- Supplementation method and frequency

### 4.6. Data Analysis

Analysis was conducted using SPSS v29.0. Descriptive statistics were applied: categorical variables were expressed as percentages, while continuous variables were expressed as means ± standard deviation for normally distributed data or as percentiles for non-parametric data.

Student’s *t*-test or ANOVA was used for comparing continuous variables. For non-normal distributions, the Kruskal–Wallis test was applied. Categorical variables were compared using the chi-square test or Fisher’s exact test. Logistic regression models were used to determine the influence of drug interactions with VD supplementation on achieving adequate serum 25(OH)D levels. A *p*-value < 0.050 was considered statistically significant.

### 4.7. Ethic Approval

This study was conducted according to the guidelines of the Declaration of Helsinki and approved by the Research Ethical Committee of the Granada Centre (0967N23).

## 5. Conclusions

VD supplementation for 12 months significantly improved serum 25(OH)D concentrations and self-perceived QoL in a population with high baseline prevalence of deficiency and multiple comorbidities. The response to supplementation was influenced by concomitant medication use: while most drug users benefited, patients receiving corticosteroids, NSAIDs, or vitamin K antagonists exhibited attenuated improvements, highlighting the need for closer monitoring in these groups. Individuals without comorbidities or those using PPIs were more likely to reach optimal 25(OH)D levels, whereas chronic conditions such as diabetes and hypercholesterolemia reduced the probability of adequacy.

Taken together, these findings reinforce the value of systematic screening and supplementation in at-risk populations. Personalized strategies, considering both comorbidity profile and concurrent pharmacological treatments, are crucial to optimize VDD status and maximize potential health benefits. Future clinical trials are warranted to confirm these results and to evaluate the long-term impact of VD adequacy on clinical outcomes, including bone health, metabolic control, and health benefits.

## Figures and Tables

**Table 1:** Vitamin D Supplement and drugs interaction.

Medication	Consequence of Interaction in Plasma Concentration
Antidiabetics	Decrease in VD concentration with Metformin and Thiazolidinedione
Cardiovascular diseases	- *Calcium Channel Blockers*, such as verapamil and diltiazem decrease in VD concentration but nifedipine increase in VD. - *Angiotensin-converting enzyme inhibitors*. There are doubts as to what the effect is, but studies generally show a decrease in VD concentration. - *Statin group*, there are those that increase the concentration of VD (rosuvastatin, fluvastatine) and those that decrease the concentration (simvastatin, lovastatin, and atorvastatin)
Gastrointestinal	- *Proton Pump Inhibitors (PPI)*. VD and calcium where high-dose PPI therapy is being employed, is recommended thus alleviates the effects of VD malabsorption caused by PPIs - *Histamine H2-Receptor Antagonists*. Cimetidine and ranitidine treated gastric ulcers identified no significant decrease in 25(OH)D serum concentrations
Central Nervous System	There is a significant inverse association between benzodiacepine concentration and VD. Also, an inverse relationship between the use of selective serotonin reuptake inhibitors and VD has been reported.

VD: Vitamin D.

**Table 2:** Socio-demographic data.

Variable	Basal Mean (SD)/N (%)	Adequate Mean (SD)/N (%)	Insufficient Mean (SD)/N (%)	Deficient Mean (SD)/N (%)
N	210	27 (12.8)	77 (36.6)	106 (50.6)
Age (years)	58.52 ± 14.15	61.54 ± 12.51	56.40 ± 14.83	59.32 ± 13.88
Female	165 (77.8)	21 (12.8)	55 (33.3)	89 (53.9)
Male	45 (22.2)	6 (13.3)	22 (48.9)	17 (37.8)
Employment Situation	82 (39.2)	12 (14.6)	29 (35.4)	41 (50.0)
(retired)	105 (49.5)	16 (15.2)	40 (38.1)	49 (46.7)
Education level	22 (10.9)	4 (18.2)	5 (22.7)	13 (59.1)
(University)	16 (7.9)	3 (17.8)	6 (37.5)	7 (44.7)
Diabetes (Yes)	15 (7.4)	2 (13.3)	8 (53.3)	5 (33.4)
Asthma/COPD (Yes)	59 (29.2)	8 (13.6)	17 (28.8)	34 (57.6)
Autoimmune (Yes)	96 (46.6)	14 (14.6)	31 (32.3)	51 (53.1)
Hypercholesterolemia (Yes)	19 (9.5)	1 (5.3)	6 (31.6)	12 (63.1)
Hypertension (Yes)	24 (11.9)	6 (25.0)	3 (12.5)	17 (62.5)
Thyroid (Yes)	26 (12.9)	3 (11.5)	8 (30.8)	15 (57.7)
Osteoporosis (Yes)	55 (26.1)	9 (16.4)	13 (23.6)	33 (60.0)
Depression (Yes)	32 (15.2)	2 (6.3)	17 (53.1)	13 (40.6)
Insomnia (Yes)	210	27 (12.8)	77 (36.6)	106 (50.6)
No pathologies (Yes)	58.52 ± 14.15	61.54 ± 12.51	56.40 ± 14.83	59.32 ± 13.88

SD: Standard Deviation; COPD: Chronic obstructive pulmonary disease.

**Table 3:** Changes in habits that influence vitamin D levels before and after 12 months of vitamin D supplementation.

Variable	Basal Mean (SD)/N (%)	Post Supplementation Mean (SD)/N (%)	*p*-Value
Serum vitamin D (25(OH)D)	21.28 ± 8.23	32.08 ± 12.55	<0.001 **
Quality of life (VAS 0–100)	68.63 ± 18.71	77.83 ± 18.45	<0.001 **
BMI	25.33 ± 4.21	25.21 ± 4.19	0.848
Walking at least 30 min/day	4.29 ± 2.48	4.71 ± 2.46	0.093
Outdoor sport hours	2.31 ± 2.85	3.33 ± 3.92	0.003 *
Avocado weekly	1.68 ± 2.05	2.22 ± 2.12	<0.001 **
Tuna weekly	1.88 ± 1.40	2.30 ± 1.41	0.003 *
Salmon weekly	0.82 ± 1.04	1.09 ± 1.07	0.010 *
Egg weekly	2.81 ± 1.56	3.35 ± 1.56	<0.001 **
Sardine or anchovy weekly	0.46 ± 0.93	0.56 ± 1.00	0.308
Supplemented dairy weekly	2.46 ± 3.20	1.48 ± 2.86	0.001 *
Mushroom weekly	0.81 ± 1.09	1.15 ± 1.16	0.003 *

BMI: Body Mass Index; SD: Standard Deviation. Chi-square *p* < 0.050 and *t*-Student *p* < 0.050. * *p* < 0.050 and ** *p* < 0.001.

**Table 4:** Changes in vitamin D level categories before and after 12 months of vitamin D supplementation according to the consumption of different medications.

Variable	N (%)	Adequate N (%)	Insufficient N (%)	Deficient N (%)	*p*-Value
**No drugs**	32 (15.2)				0.001 **
Basal		2 (6.3)	17 (53.1)	13 (40.6)	
Postsupplement		15 (46.9)	10 (31.3)	7 (21.8)	
**Corticosteroid**	9 (4.3)				0.661
Basal		1 (11.2)	4 (44.4)	4 (44.4)	
Postsupplement		2 (22.4)	6 (66.8)	1 (11.2)	
**Benzodiazepine**	43 (20.5)				0.006 *
Basal		5 (11.6)	11 (25.6)	27 (62.8)	
Postsupplement		8 (18.6)	13 (30.2)	22 (51.2)	
**Proton pump inhibitor**	36 (17.1)				0.042 *
Basal		3 (8.3)	12 (33.3)	21 (58.4)	
Postsupplement		11 (30.6)	20 (55.6)	5 (13.8)	
**Beta blocker**	17 (8.1)				0.009 *
Basal		0 (0.0)	6 (35.3)	11 (64.7)	
Postsupplement		8 (47.1)	8 (47.1)	1 (5.8)	
**NSAID**	8 (3.8)				0.439
Basal		1 (12.5)	1 (12.5)	6 (75.0)	
Postsupplement		1 (12.5)	4 (50.0)	3 (37.5)	
**Levothyroxine**	12 (5.7)				0.003 *
Basal		1 (8.3)	3 (25.0)	8 (66.7)	
Postsupplement		8 (66.7)	3 (25.0)	1 (8.3)	
**Vitamin K antagonists**	8 (3.8)				0.854
Basal		1 (12.5)	3 (37.5)	4 (50.0)	
Postsupplement		4 (50.0)	3 (37.5)	1 (12.5)	
**Biguanide**	9 (4.3)				0.039 *
Basal		1 (11.2)	4 (44.4)	4 (44.4)	
Postsupplement		5 (55.5)	3 (33.3)	1 (11.2)	
**Inhaler asthma/COPD**	8 (3.8)				0.986
Basal		1 (12.5)	3 (37.5)	4 (50.0)	
Postsupplement		1 (12.5)	4 (50.0)	3 (37.5)	
**Xanthine oxidase inhibitor**	10 (4.8)				0.020 *
Basal		0 (0.0)	4 (40.0)	6 (60.0)	
Postsupplement		7 (70.0)	3 (30.0)	0 (0.0)	
**Ezetimibe**	10 (4.8)				0.979
Basal		0 (0.0)	4 (40.0)	6 (60.0)	
Postsupplement		6 (60.0)	3 (30.0)	1 (10.0)	
**Statin**	39 (18.6)				0.003 *
Basal		4 (10.2)	11 (28.2)	24 (61.6)	
Postsupplement		24 (61.6)	10 (25.6)	5 (12.8)	
**Diuretic**	20 (9.5)				0.114
Basal		5 (25.0)	6 (30.0)	9 (45.0)	
Postsupplement		9 (45.0)	10 (50.0)	1 (5.0)	
**Serotonin reuptake inhibitor**	9 (4.3)				0.800
Basal		1 (11.2)	4 (44.4)	4 (44.4)	
Postsupplement		4 (44.4)	2 (22.2)	3 (33.3)	
**Antagonists of the angiotensin II**	14 (6.7)				0.972
Basal		4 (28.6)	5 (35.7)	5 (35.7)	
Postsupplement		5 (35.7)	6 (42.9)	3 (21.4)	
**ACE inhibitor**	27 (12.9)				0.032 *
Basal		2 (7.4)	8 (29.6)	16 (59.3)	
Postsupplement		13 (48.2)	10 (37.0)	4 (14.8)	
**Acetaminophen**	25 (11.9)				0.540
Basal		4 (16.0)	5 (20.0)	16 (64.0)	
Postsupplement		9 (36.0)	9 (36.0)	7 (28.0)	
**Insulin**	5 (2.4)				0.800
Basal		2 (40.0)	0 (0.0)	3 (60.0)	
Postsupplement		3 (60.0)	1 (20.0)	1 (20.0)	
**Calcium channel blocker**	19 (9.0)				0.586
Basal		0 (0.0)	9 (47.4)	10 (52.6)	
Postsupplement		6 (31.5)	9 (47.4)	4 (21.1)	
**Antihistamine**	14 (6.7)				0.092
Basal		0 (0.0)	7 (50.0)	7 (50.0)	
Postsupplement		4 (28.6)	7 (50.0)	3 (21.4)	

COPD: Chronic obstructive pulmonary disease; NSAID: Non-Steroidal Anti-Inflammatory Drugs; ACE: Angiotensin-Converting Enzyme. Chi-square *p* < 0.050. * *p* < 0.050 and ** *p* < 0.001.

**Table 5:** Differences in vitamin D levels and quality of life among consumers of different medications before and after 12 months of vitamin D supplementation.

	Vit D Pre	Vit D Post	QoL-VAS Pre	QoL-VAS Post
No drugs	21.35 ± 7.17	30.11 ± 11.97 *	79.24 ± 15.06	89.06 ± 11.53 *
**Benzodiazepine**	19.82 ± 7.48	32.41 ± 16.10 **	61.10 ± 17.05	70.96 ± 19.19 *
Other drugs	21.61 ± 8.63	32.54 ± 12.80 **	68.93 ± 18.89	75.18 ± 19.91 *
BZ vs. Other drugs	0.240	0.965	**0.013**	0.213
Other drugs vs. No drugs	0.884	0.406	**0.004**	**0.000**
BZ vs. No drugs	0.395	0.560	**0.000**	**0.000**
**Proton pump inhibitor**	21.16 ± 6.68	32.13 ± 17.28 *	62.61 ± 21.95	69.67 ± 18.57
Other drugs	21.04 ± 8.82	32.62 ± 12.44 **	68.28 ± 12.34	75.62 ± 20.00 *
PPI vs. Other drugs	0.940	0.867	0.077	0.081
Other drugs vs. No drugs	0.863	0.378	**0.001**	**0.000**
PPI vs. No drugs	0.913	0.631	**0.000**	**0.000**
**Beta-blocker**	19.35 ± 5.48	30.30 ± 8.02 **	57.17 ± 20.16	69.78 ± 21.66 *
Other drugs	21.31 ± 8.61	32.86 ± 14.39 **	68.24 ± 18.08	74.68 ± 19.43 *
BB vs. Other drugs	0.365	0.477	**0.008**	0.270
Other drugs vs. No drugs	0.979	0.387	**0.001**	**0.000**
BB vs. No drugs	0.329	0.955	**0.000**	**0.000**
**NSAID**	20.08 ± 7.40	21.80 ± 3.20	61.00 ± 19.41	67.00 ± 23.00
Other drugs	21.13 ± 8.38	32.77 ± 13.74 **	67.15 ± 18.64	74.45 ± 19.53 **
NSAID vs. Other drugs	0.728	0.171	0.314	0.248
Other drugs vs. No drugs	0.897	0.379	**0.001**	**0.000**
NSAID vs. No drugs	0.661	0.250	**0.003**	**0.000**
**Levothyroxine**	20.18 ± 6.53	32.59 ± 9.10 *	70.59 ± 15.09	73.53 ± 19.83
Other drugs	21.15 ± 8.47	32.49 ± 14.16 **	66.39 ± 19.03	74.07 ± 19.80 *
LT vs. Other drugs	0.700	0.980	0.380	0.915
Other drugs vs. No drugs	0.908	0.446	**0.000**	**0.000**
LT vs. No drugs	0.630	0.520	0.060	**0.001**
**Vitamin K antagonists**	22.40 ± 6.95	31.91 ± 11.88	72.73 ± 11.55	70.00 ± 21.91
Other drugs	20.99 ± 8.40	32.54 ± 13.84 **	66.40 ± 18.85	74.29 ± 19.64 *
VKA vs. Other drugs	0.564	0.899	0.366	0.872
Other drugs vs. No drugs	0.827	0.426	**0.000**	**0.000**
VKA vs. No drugs	0.627	0.716	0.197	**0.003**
**Biguanide**	22.59 ± 4.81	37.70 ± 14.83 *	58.50 ± 25.17	68.00 ± 21.50
Other drugs	20.97 ± 8.50	32.28 ± 13.65 **	67.30 ± 18.20	74.39 ± 19.65 *
Biguanide vs. Other drugs	0.573	0.388	0.149	0.322
Other drugs vs. No drugs	0.824	0.472	**0.001**	**0.000**
Biguanide vs. No drugs	0.632	0.225	**0.002**	**0.000**
**Inhalers for asthma/COPD**	21.56 ± 7.64	23.97 ± 6.14	65.00 ± 13.54	72.00 ± 23.94
Other drugs	21.04 ± 8.37	32.72 ± 13.76 **	66.90 ± 18.98	74.14 ± 19.54 *
Inhaler vs. Other drugs	0.864	0.276	0.755	0.74
Other drugs vs. No drugs	0.856	0.390	**0.001**	**0.000**
Inhaler vs. No drugs	0.943	0.396	**0.011**	**0.003**
**Xanthine oxidase inhibitor**	19.49 ± 5.35	30.80 ± 7.56 *	66.79 ± 16.24	70.00 ± 22.36
Other drugs	21.19 ± 8.49	32.67 ± 14.16 **	66.80 ± 18.93	74.35 ± 19.56*
XOI vs. Other drugs	0.535	0.666	0.998	0.447
Other drugs vs. No drugs	0.926	0.412	**0.000**	**0.000**
XOI vs. No drugs	0.460	0.864	**0.015**	**0.000**
**Ezetimibe**	22.30 ± 7.95	33.05 ± 12.57	69.00 ± 21.45	77.50 ± 25.30
Other drugs	21.00 ± 8.35	32.47 ± 13.79 **	66.66 ± 18.57	73.80 ± 19.43 *
Ezetimibe vs. Other drugs	0.669	0.914	0.702	0.567
Other drugs vs. No drugs	0.835	0.439	**0.000**	**0.000**
Ezetimibe vs. No drugs	0.749	0.576	0.096	**0.047**
**Statin**	20.43 ± 8.45	33.07 ± 15.42 **	68.46 ± 18.71	77.87 ± 18.19 *
Other drugs	21.33 ± 8.28	32.22 ± 12.83 **	66.04 ± 18.71	72.24 ± 20.26 *
Statin vs. Other drugs	0.565	0.749	0.429	0.083
Other drugs vs. No drugs	0.987	0.475	**0.000**	**0.000**
Statin vs. No drugs	0.638	0.424	**0.006**	**0.002**
**Diuretic**	26.03 ± 11.34	29.67 ± 6.04	56.96 ± 20.82	61.27 ± 27.32
Other drugs	20.26 ± 7.45	32.97 ± 14.52 **	68.28 ± 17.96	75.90 ± 17.73 **
Diuretic vs. Other drugs	**0.004**	0.358	**0.006**	**0.001**
Other drugs vs. No drugs	0.482	0.373	**0.001**	**0.000**
Diuretic vs. No drugs	0.087	0.888	**0.000**	**0.000**
**Corticosteroid**	27.90 ± 7.90	28.36 ± 10.16	72.00 ± 15.31	74.50 ± 26.29
Other drugs	20.82 ± 8.24	32.76 ± 18.86 **	66.48 ± 18.86	73.99 ± 19.38 **
CC vs. Other drugs	0.061	0.411	0.366	0.937
Other drugs vs. No drugs	0.752	0.387	**0.000**	**0.000**
CC vs. No drugs	0.073	0.728	0.192	**0.016**
**Serotonin reuptake inhibitor**	20.31 ± 7.45	27.70 ± 14.04	65.00 ± 17.16	76.50 ± 13.34
Other drugs	21.12 ± 8.38	32.75 ± 13.68 **	66.90 ± 18.82	73.86 ± 20.10 *
SRI vs. Other drugs	0.790	0.38	0.755	0.683
Other drugs vs. No drugs	0.890	0.382	**0.000**	**0.000**
SRI vs. No drugs	0.721	0.672	**0.015**	**0.006**
**Angiotensin II antagonist**	25.73 ± 13.21	28.28 ± 11.87	62.00 ± 21.73	70.40 ± 26.30
Other drugs	20.56 ± 7.49	33.15 ± 13.87 **	67.41 ± 18.25	74.50 ± 18.77 *
AIIA vs. Other drugs	**0.027**	0.186	0.224	0.385
Other drugs vs. No drugs	0.611	0.324	**0.001**	**0.000**
AIIA vs. No drugs	0.170	0.636	**0.001**	**0.001**
**ACEI**	21.20 ± 7.87	31.85 ± 12.46 *	64.94 ± 17.64	68.94 ± 21.71
Other drugs	21.04 ± 8.44	32.67 ± 14.04 **	67.24 ± 18.96	75.23 ± 19.13 *
ACEI vs. Other drugs	0.931	0.790	0.521	0.100
Other drugs vs. No drugs	0.858	0.413	**0.001**	**0.000**
ACEI vs. No drugs	0.939	0.621	**0.001**	**0.000**
**Acetaminophen**	20.16 ± 10.94	28.09 ± 14.12 *	60.89 ± 21.48	62.15 ± 27.05
Other drugs	21.27 ± 7.68	33.22 ± 13.54 **	67.91 ± 17.97	76.24 ± 17.29 **
Acetaminophen vs. Other drugs	0.545	0.152	0.068	**0.001**
Other drugs vs. No drugs	0.958	0.302	**0.001**	**0.000**
Acetaminophen vs. No drugs	0.636	0.623	**0.000**	**0.000**
**Insulin**	23.78 ± 9.52	30.04 ± 10.20	71.43 ± 26.88	83.57 ± 19.30
Other drugs	20.97 ± 8.29	32.63 ± 13.86 **	66.60 ± 18.36	73.61 ± 19.72 *
Insulin vs. Other drugs	0.459	0.652	0.505	0.192
Other drugs vs. No drugs	0.822	0.409	**0.000**	**0.000**
Insulin vs. No drugs	0.510	0.729	0.289	**0.000**
**Calcium channel blockers**	20.79 ± 5.56	28.92 ± 11.39 *	55.96 ± 24.07	65.33 ± 20.29
Other drugs	21.12 ± 8.67	33.13 ± 13.99 **	68.51 ± 17.17	75.44 ± 19.36 *
CCB vs. Other drugs	0.875	0.230	**0.002**	**0.020**
Other drugs vs. No drugs	0.893	0.332	**0.001**	**0.000**
CCB vs. No drugs	0.776	0.928	**0.000**	**0.000**
**Antihistamine**	19.21 ± 5.42	29.73 ± 8.36 *	80.00 ± 13.16	82.31 ± 13.17
Other drugs	21.28 ± 8.56	32.65 ± 13.91 **	65.65 ± 18.68	73.34 ± 20.07 **
Antihistamine vs. Other drugs	0.378	0.613	0.006	0.116
Other drugs vs. No drugs	0.965	0.408	**0.000**	**0.000**
Antihistamine vs. No drugs	0.330	0.942	0.871	0.093

BZ: Benzodiazepine; PPI: Proton pump inhibitor; BB: Beta-blocker; LT: Levothyroxine; VKA: Vitamin K antagonists; CC: Corticosteroid; SRI: Serotonin reuptake inhibitor; AIIA: Angiotensin II antagonist; COPD: Chronic obstructive pulmonary disease; NSAID: Non-Steroidal Anti-Inflammatory Drugs; ACEI: Angiotensin-Converting Enzyme Inhibitors; CCB: Calcium channel blockers. *t*-Student *p* < 0.050. * *p* < 0.050 and ** *p* < 0.001. Bold values *p* < 0.050.

**Table 6:** Vitamin D levels and habits.

Adequate Vitamin D	Baseline		Post Supplementation	
	OR	95% CI	OR	95% CI
Adherence to VD	2.57	0.98–6.62	**15.31**	**2.90–80.75**
Gender (female)	1.43	0.61–3.40	1.14	0.47–2.74
BMI	1.69	0.74–3.90	1.11	0.56–2.23
PPI	1.19	0.36–3.95	**1.83**	**1.04–3.23**
Beta Blocker	**0.89**	**0.85–0.94**	0.90	0.56–1.46
NSAID	1.04	0.79–1.35	**0.47**	**0.40–0.56**
Inhaler asthma/COPD	1.04	0.79–1.35	**0.47**	**0.40–0.56**
Xanthine oxidase inhibitor	**0.89**	**0.85–0.95**	1.35	0.48–3.02
Diuretic	**4.12**	**1.26–13.47**	1.59	0.76–3.33
Corticosteroid	1.37	0.78–2.42	**0.62**	**0.40–0.97**
AAIIR	**4.70**	**1.28–17.26**	0.52	0.18–1.52
Insulin	**7.05**	**1.09–45.78**	0.93	0.18–4.77
Calcium channel blockers	**0.89**	**0.84–0.94**	1.63	0.83–3.19
Antihistamine	**0.90**	**0.85–0.95**	0.71	0.39–1.29
Diabetes	1.09	0.28–4.32	**0.20**	**0.06–0.67**
Hypercholesterolemia	0.77	0.25–2.36	**0.25**	**0.09–0.69**
No pathologies	0.53	0.12–2.42	**4.00**	**1.29–11.51**

OR: Odds Ratio; CI: Confidence Interval. Adequate Vitamin D: level of serum vitamin D higher than 30 ng/mL. Chi-Square *p* < 0.050. Bold value *p* < 0.050.

## Data Availability

The original contributions presented in this study are included in the article. Further inquiries can be directed to the corresponding author.

## Abbreviations

The following abbreviations are used in this manuscript:

25(OH)D	25-hydroxyvitamin D
ACE	Angiotensin-converting enzyme
BMI	Body Mass Index
NSAID	Nonsteroidal anti-inflammatory drug
PPI	Proton Pump Inhibitor
QoL	Quality of Life
VDD	Vitamin D Deficiency

## References

[1] Bell T.D., Demay M.B., Burnett-Bowie S.A. (2010). The biology and pathology of vitamin D control in bone. J. Cell. Biochem..

[2] Goltzman D. (2018). Functions of vitamin D in bone. Histochem. Cell Biol..

[3] Gembillo G., Cernaro V., Siligato R., Curreri F., Catalano A., Santoro D. (2020). Protective Role of Vitamin D in Renal Tubulopathies. Metabolites.

[4] Battistini C., Ballan R., Herkenhoff M.E., Saad S.M.I., Sun J. (2020). Vitamin D Modulates Intestinal Microbiota in Inflammatory Bowel Diseases. Int. J. Mol. Sci..

[5] Patel H., Gupta V., Jain K., Yagnik P., Nair N.M.S., Reddy G.R.A. (2025). Oral Versus Injectable Vitamin D Therapy for Treating Nutritional Rickets in Indian Children: A Comparative Study. Indian J. Orthop..

[6] Ma M., Zhang Y., Liu J., Tian C., Duan Z., Huang X., Geng B. (2025). Associations of the serum 25-hydroxyvitamin D with mortality among patients in osteopenia or osteoporosis. Bone.

[7] Bouillon R., Schuit F., Antonio L., Rastinejad F. (2019). Vitamin D Binding Protein: A Historic Overview. Front. Endocrinol..

[8] Zmijewski M.A., Carlberg C. (2020). Vitamin D receptor(s): In the nucleus but also at membranes?. Exp. Dermatol..

[9] Al-Ishaq R.K., Kubatka P., Brozmanova M., Gazdikova K., Caprnda M., Busselberg D. (2021). Health implication of vitamin D on the cardiovascular and the renal system. Arch. Physiol. Biochem..

[10] Pittas A.G., Lau J., Hu F.B., Dawson-Hughes B. (2007). The role of vitamin D and calcium in type 2 diabetes. A systematic review and meta-analysis. J. Clin. Endocrinol. Metab..

[11] Lei M., Liu Z., Guo J. (2020). The Emerging Role of Vitamin D and Vitamin D Receptor in Diabetic Nephropathy. BioMed Res. Int..

[12] Manson J.E., Cook N.R., Lee I.M., Christen W., Bassuk S.S., Mora S., Gibson H., Gordon D., Copeland T., D’Agostino D. (2019). Vitamin D Supplements and Prevention of Cancer and Cardiovascular Disease. N. Engl. J. Med..

[13] Gnagnarella P., Raimondi S., Aristarco V., Johansson H.A., Bellerba F., Corso F., Gandini S. (2020). Vitamin D Receptor Polymorphisms and Cancer. Adv. Exp. Med. Biol..

[14] Mocanu V., Oboroceanu T., Zugun-Eloae F. (2013). Current status in vitamin D and regulatory T cells--immunological implications. Rev. Med. Chir. Soc. Med. Nat. Iaşi.

[15] Lips P., Cashman K.D., Lamberg-Allardt C., Bischoff-Ferrari H.A., Obermayer-Pietsch B., Bianchi M.L., Stepan J., El-Hajj Fuleihan G., Bouillon R. (2019). Current vitamin D status in European and Middle East countries and strategies to prevent vitamin D deficiency: A position statement of the European Calcified Tissue Society. Eur. J. Endocrinol..

[16] Haitchi S., Moliterno P., Widhalm K. (2023). Prevalence of vitamin D deficiency in seniors—A retrospective study. Clin. Nutr. ESPEN.

[17] Cui A., Zhang T., Xiao P., Fan Z., Wang H., Zhuang Y. (2023). Global and regional prevalence of vitamin D deficiency in population-based studies from 2000 to 2022: A pooled analysis of 7.9 million participants. Front. Nutr..

[18] Argano C., Torres A., Orlando V., Cangialosi V., Maggio D., Pollicino C., Corrao S. (2025). Molecular Insight into the Role of Vitamin D in Immune-Mediated Inflammatory Diseases. Int. J. Mol. Sci..

[19] Oh T.R., Kim C.S., Bae E.H., Ma S.K., Han S.H., Sung S.A., Hyubeck L., Kook H.O., Curie A., Soo W.K. (2017). Association between vitamin D deficiency and health-related quality of life in patients with chronic kidney disease from the KNOW-CKD study. PLoS ONE.

[20] Akyuz G., Sanal-Toprak C., Yagci I., Giray E., Kuru-Bektasoglu P. (2017). The effect of vitamin D supplementation on pain, quality of life, and nerve conduction studies in women with chronic widespread pain. Int. J. Rehabil. Res..

[21] Ramasamy I. (2020). Vitamin D Metabolism and Guidelines for Vitamin D Supplementation. Clin. Biochem. Rev..

[22] Beauchet O., Cooper-Brown L.A., Allali G. (2021). Vitamin D Supplementation and Cognition in Adults: A Systematic Review of Randomized Controlled Trials. CNS Drugs.

[23] Gastelurrutia M.A., Faus M.J., Fernandez-Llimos F. (2005). Providing patient care in community pharmacies in Spain. Ann. Pharmacother..

[24] Amador-Fernandez N., Gastelurrutia M.A., Garcia-Cardenas V. (2023). Development of self-care in Spanish community pharmacies. Explor. Res. Clin. Soc. Pharm..

[25] Fikri-Benbrahim N., Garcia-Cardenas V., Saez-Benito L., Gastelurrutia M.A., Faus M.P., Schneider M.P., Aslani P. (2009). Adherence: A review of education, research, practice and policy in Spain. Pharm. Pract..

[26] Valverde-Merino M.I., Martinez-Martinez F., Garcia-Mochon L., Benrimoj S.I., Malet-Larrea A., Perez-Escamilla B., Zarzuelo M.J., Torres-Robles A., Gastelurrutia M.A., Varas-Doval R. (2021). Cost-Utility Analysis of a Medication Adherence Management Service Alongside a Cluster Randomized Control Trial in Community Pharmacy. Patient Prefer. Adherence.

[27] Llorca C.V.Y., Cortes Castell E., Ribera Casado J.M., de Lucas Ramos P., Casteig Ayestaran J.L., Casteig Blanco A., Gil Guillen V.F., Rizo Baeza M. (2021). Factors Associated with Non-Adherence to Drugs in Patients with Chronic Diseases Who Go to Pharmacies in Spain. Int. J. Environ. Res. Public Health.

[28] Berenbrok L.A., Tang S., Gabriel N., Guo J., Sharareh N., Patel N., Dickson S., Hernandez I. (2022). Access to community pharmacies: A nationwide geographic information systems cross-sectional analysis. J. Am. Pharm. Assoc. JAPhA.

[29] Aguilar Shea A.L., Munoz Moreno-Arrones O., Palacios Martinez D., Vano-Galvan S. (2020). Vitamin D for daily practice. Semergen.

[30] Syrnyk M., Glass B. (2023). Pharmacist interventions in medication adherence in patients with mental health disorders: A scoping review. Int. J. Pharm. Pract..

[31] Wilhelmsen N.C., Eriksson T. (2019). Medication adherence interventions and outcomes: An overview of systematic reviews. Eur. J. Hosp. Pharm. Sci. Pract..

[32] Presley B., Groot W., Pavlova M. (2019). Pharmacy-led interventions to improve medication adherence among adults with diabetes: A systematic review and meta-analysis. Res. Soc. Adm. Pharm. RSAP.

[33] Wakeman M. (2021). A Literature Review of the Potential Impact of Medication on Vitamin D Status. Risk Manag. Healthc. Policy.

[34] Robien K., Oppeneer S.J., Kelly J.A., Hamilton-Reeves J.M. (2013). Drug-vitamin D interactions: A systematic review of the literature. Nutr. Clin. Pract..

[35] Pilz S., Marz W., Cashman K.D., Kiely M.E., Whiting S.J., Holick M.F., Grant W.B., Pludowski P., Hiligsmann M., Trummer C. (2018). Rationale and Plan for Vitamin D Food Fortification: A Review and Guidance Paper. Front. Endocrinol..

[36] Zhang J., Zhu Z., Niu Y., Cao Z.B. (2024). Exercise combined with vitamin D supplementation has additive health effects on short physical performance battery and stair climbing in older adults: A scope review of randomised controlled trials. Br. J. Nutr..

[37] Rusu M.E., Bigman G., Ryan A.S., Popa D.S. (2024). Investigating the Effects and Mechanisms of Combined Vitamin D and K Supplementation in Postmenopausal Women: An Up-to-Date Comprehensive Review of Clinical Studies. Nutrients.

[38] Bo Y., Liu C., Ji Z., Yang R., An Q., Zhang X., You J., Duan D., Sun Y., Zhu Y. (2019). A high whey protein, vitamin D and E supplement preserves muscle mass, strength, and quality of life in sarcopenic older adults: A double-blind randomized controlled trial. Clin. Nutr..

[39] Davoudi M., Allame Z., Niya R.T., Taheri A.A., Ahmadi S.M. (2021). The synergistic effect of vitamin D supplement and mindfulness training on pain severity, pain-related disability and neuropathy-specific quality of life dimensions in painful diabetic neuropathy: A randomized clinical trial with placebo-controlled. J. Diabetes Metab. Disord..

[40] Hoffmann M.R., Senior P.A., Mager D.R. (2015). Vitamin D supplementation and health-related quality of life: A systematic review of the literature. J. Acad. Nutr. Diet..

[41] Qi S., Zhao M., Sun Y., Boro S., Arora B., Rastogi S. (2025). Impact of vitamin D supplementation on symptom severity and quality of life in patients with irritable bowel syndrome: A meta-analysis. Adv. Clin. Exp. Med..

[42] Li X., Liu Y., Zheng Y., Wang P., Zhang Y. (2018). The Effect of Vitamin D Supplementation on Glycemic Control in Type 2 Diabetes Patients: A Systematic Review and Meta-Analysis. Nutrients.

[43] Alqadi A., Almodyan E., Alanazi M.N., Alghamdi S.S., Ahmed M.H., Badi S., Alghamdi A.S. (2025). Vitamin D supplements and effect on glycemic control and lipid profile in individuals living with diabetes: A retrospective study. Eur. Rev. Med. Pharmacol. Sci..

[44] Loke Y.K., Gilbert D., Thavarajah M., Blanco P., Wilson A.M. (2015). Bone mineral density and fracture risk with long-term use of inhaled corticosteroids in patients with asthma: Systematic review and meta-analysis. BMJ Open.

[45] Rejnmark L., Vestergaard P., Pedersen A.R., Heickendorff L., Andreasen F., Mosekilde L. (2003). Dose-effect relations of loop- and thiazide-diuretics on calcium homeostasis: A randomized, double-blinded Latin-square multiple cross-over study in postmenopausal osteopenic women. Eur. J. Clin. Investig..

[46] Sathyapalan T., Shepherd J., Arnett C., Coady A.M., Kilpatrick E.S., Atkin S.L. (2010). Atorvastatin increases 25-hydroxy vitamin D concentrations in patients with polycystic ovary syndrome. Clin. Chem..

[47] Sathyapalan T., Shepherd J., Atkin S.L., Kilpatrick E.S. (2013). The effect of atorvastatin and simvastatin on vitamin D, oxidative stress and inflammatory marker concentrations in patients with type 2 diabetes: A crossover study. Diabetes Obes. Metab..

[48] Perez-Castrillon J.L., Vega G., Abad L., Sanz A., Chaves J., Hernandez G., Duenas A. (2007). Effects of Atorvastatin on vitamin D levels in patients with acute ischemic heart disease. Am. J. Cardiol..

[49] Grober U., Kisters K. (2012). Influence of drugs on vitamin D and calcium metabolism. Dermato-Endocrinology.

[50] Vogt S., Decke S., de Las Heras Gala T., Linkohr B., Koenig W., Ladwig K.H., Peters A., Thorand B. (2015). Prospective association of vitamin D with frailty status and all-cause mortality in older adults: Results from the KORA-Age Study. Prev. Med..

[51] Perez-Castrillon J.L., Justo I., Sanz A., De Luis D., Duenas A. (2006). Effect of angiotensin converting enzyme inhibitors on 1.25-(OH)2 D levels of hypertensive patients. Relationship with ACE polymorphisms. Horm. Metab. Res..

[52] Stenova E., Steno B., Killinger Z., Baqi L., Payer J. (2011). Effect of long-term oral anticoagulant therapy on bone mineral density and bone turnover markers: A prospective 12 month study. Bratisl. Lek. Listy.

[53] Sawicka-Powierza J., Konstantynowicz J., Jablonska E., Zelazowska-Rutkowska B., Jelski W., Abramowicz P., Sasinowski C., Chlabicz S. (2018). The Association Between Long-Term Acenocoumarol Treatment and Vitamin D Deficiency. Front. Endocrinol..

[54] Verhoeven V., Vanpuyenbroeck K., Lopez-Hartmann M., Wens J., Remmen R. (2012). Walk on the sunny side of life--epidemiology of hypovitaminosis D and mental health in elderly nursing home residents. J. Nutr. Health Aging.

[55] van Orten-Luiten A.C., Janse A., Dhonukshe-Rutten R.A., Witkamp R.F. (2016). Vitamin D deficiency as adverse drug reaction? A cross-sectional study in Dutch geriatric outpatients. Eur. J. Clin. Pharmacol..

[56] Chan V., Cave A.J., Banh H.L. (2015). Self-reported osteoporosis prevention in inhaled corticosteroid users in community pharmacy setting. SAGE Open Med..

[57] Gardner D.G., Chen S., Glenn D.J. (2013). Vitamin D and the heart. Am. J. Physiol. Regul. Integr. Comp. Physiol..

[58] Wimalawansa S.J. (2018). Vitamin D and cardiovascular diseases: Causality. J. Steroid Biochem. Mol. Biol..

[59] Muscogiuri G., Sorice G.P., Ajjan R., Mezza T., Pilz S., Prioletta A., Scragg R., Volpe S.L., Witham M.D., Giaccari A. (2012). Can vitamin D deficiency cause diabetes and cardiovascular diseases? Present evidence and future perspectives. Nutr. Metab. Cardiovasc. Dis. NMCD.

[60] Malaeb D., Hallit S., Salameh P. (2017). Assessment of vitamin D levels, awareness among Lebanese pharmacy students, and impact of pharmacist counseling. J. Epidemiol. Glob. Health.

[61] Broussard D.L. (2013). Public health in pharmacy: Improving vitamin D status in the U.S. population. J. Am. Pharm. Assoc..

[62] Teramura-Gronblad M., Hosia-Randell H., Muurinen S., Pitkala K. (2010). Use of proton-pump inhibitors and their associated risks among frail elderly nursing home residents. Scand. J. Prim. Health Care.

[63] Balestroni G., Bertolotti G. (2012). EuroQol-5D (EQ-5D): An instrument for measuring quality of life. Monaldi Arch. Chest Dis..

[64] Lopez-Carmona F., Toro-Ruiz A., Gomez-Guzman M., Valverde-Merino M.I., Piquer-Martinez C., Zarzuelo M.J. (2023). Community pharmacy is the key to improving vitamin D levels. Explor. Res. Clin. Soc. Pharm..

